# The angiotensin II AT1 receptor-associated protein Arap1 is involved in sepsis-induced hypotension

**DOI:** 10.1186/cc12809

**Published:** 2013-07-11

**Authors:** Katharina Mederle, Frank Schweda, Veronika Kattler, Elisabeth Doblinger, Keishi Miyata, Klaus Höcherl, Yuichi Oike, Hayo Castrop

**Affiliations:** 1 Institute of Physiology, University of Regensburg, Regensburg, Germany; 2 Department of Molecular Genetics, Kumamoto University, Kumamoto, Japan

## Abstract

**Introduction:**

Hypotension in septic patients results from hypovolemia, vasodilatation and hyporeactivity to vasoconstrictors, such as angiotensin II. The AT1 receptor-associated protein 1 (Arap1) is expressed in vascular smooth muscle cells and increases the surface expression of the AT1-receptor *in vitro*. We hypothesized that dysregulation of Arap1 may contribute to vascular hyporeactivity to angiotensin II during endotoxemia.

**Methods:**

Arap1-deficient mice were used to assess the role of Arap1 in sepsis-induced hypotension. The isolated perfused kidney was used as an *in vitro *model to determine the relevance of Arap1 for vascular resistance and sensitivity to angiotensin II.

**Results:**

During endotoxemia, mean arterial blood pressure (MAP) decreased in both genotypes, with the time course of sepsis-induced hypotension being markedly accelerated in Arap1-/- compared to +/+ mice. However, baseline MAP was similar in Arap1-/- and wildtype mice (102 ± 2 vs.103 ± 2 mmHg; telemetry measurements; *n *= 10; *P *= 0.66). Following lipopolysaccharide (LPS) injections (3 mg/kg), Arap1 expression was successively down-regulated in the wildtype mice, reaching levels below 10% of baseline expression. The endotoxemia-related decline in Arap1 expression could be recapitulated in cultured mesangial cells by incubation with pro-inflammatory cytokines, such as tumor necrosis factor α and interferon γ. Plasma renin concentration was increased in Arap1-/- mice compared to wildtype mice (66 ± 6 vs. 41 ± 4 ng AngI/ml/h; *n *= 23; *P *= 0.001), presumably contributing to preserved MAP under baseline conditions. The sensitivity of the vasculature to angiotensin II was reduced in Arap1-/- compared to +/+ mice, as determined in the isolated perfused kidney.

**Conclusions:**

Our data suggest that down-regulation of Arap1 expression during sepsis contributes to the development of hypotension by causing reduced vascular sensitivity to angiotensin II.

## Introduction

Circulatory failure is the most common and fatal hallmark of sepsis in critically ill patients. The key hemodynamic characteristic of sepsis is arterial vasodilation associated with a decrease in total vascular resistance. Hypotension in septic patients results from both hypovolemia and inadequate vasodilatation [1]. Typically, septic patients show marked hyporeactivity to both endogenously generated and exogenously administered vasoconstrictors [1,2]. The resistance to vasoconstrictors includes otherwise potent factors such as norepinephrine, endothelins, and angiotensin II. Down-regulation of the corresponding receptors, such as adrenergic α-receptors, ET_A _receptors and angiotensin AT1 receptors, is involved in the functional resistance to vasopressor hormones. Furthermore, because the concentrations of catecholamines, endothelins and angiotensin II are markedly elevated during sepsis, their corresponding receptors may be desensitized [3-6]. Additionally, the excess presence of vasodilatory substances, such as nitric oxide and prostanoids, may cause the maximum dilatation of resistance vessels and may exceed the regulatory range of vascular smooth muscle cells, which is usually determined by the balanced presence of vasoconstrictors and vasodilators [7].

Diminished vasoconstrictor function may be related to reduced receptor transcription, translation and/or membrane trafficking [8-13]. For the latter, the involvement of receptor-associated proteins might be relevant, including the recently discovered angiotensin II AT1 receptor-associated proteins [8,14]. Several different receptor-associated proteins have been described for the AT1 receptor, which is the most relevant mediator of the renin angiotensin system (RAS). Among these, the AT1-associated protein Arap1 (AT1 receptor-associated protein 1) appears to be most relevant because it is expressed predominantly in the vasculature of many organs, overlapping in location with AT1 receptors [15-18]. Arap1 supports the trafficking of the AT1 receptor to the cell membrane, leading to locally enhanced sensitivity [17]. Furthermore, the expression of Arap1 is regulated in a manner such that the presence of angiotensin II results in a marked down-regulation of Arap1, establishing a local negative feedback loop by reducing the vasculature's sensitivity when angiotensin II concentrations rise [18].

In the present study, we used Arap1-deficient mice to address the hypothesis that Arap1 might be involved in the vasculature's loss of sensitivity to angiotensin II during sepsis.

We found that during lipopolysaccharide (LPS)-induced sepsis in mice, Arap1 expression was markedly down-regulated; Arap1 expression was similarly reduced in cultured cells in the presence of pro-inflammatory cytokines. The blood pressure homeostasis during endotoxemia was more severely compromised in Arap1-deficient mice than in wildtypes, suggesting that down-regulation of Arap1 expression during the course of sepsis may contribute to the development of hyporeactivity to angiotensin II.

## Methods

### Animal experiments

Six- to nine-week-old male Arap1-/- and wildtype littermates derived from heterozygous breeding pairs were used in the experiments [19]. Unless otherwise noted, the animals had free access to standard rodent chow and tap water. The animals were housed with a 12 hour:12 hour day:night cycle. All animal experiments were approved by local ethics committees (*Regierung der Oberpfalz *for the University of Regensburg and the Animal Care Committee of Kumamoto University) and performed according to the National Institutes of Health's *Guidelines for the Use of Laboratory Animals*. In addition to baseline measurements, the following experimental protocol were used: (i) animals received the angiotensin converting enzyme (ACE) inhibitor enalapril in their drinking water (10 mg/kg/d), and (ii) to induce sepsis, mice received a single *i.p*. injection of lipopolysaccharide (LPS, Sigma-Aldrich, Munich, Germany, 3 mg/kg).

### Blood pressure measurement by radio-telemetry

The Data Sciences International telemetry system (St. Paul, MN, USA) was used for the experiments. Transmitters (model TA11PA-C10) were magnetically activated >24 hours before implantation. The mice were anesthetized with ketamine and xylazine (90 and 10 mg/kg, respectively), and the telemeter catheter was inserted into the left carotid artery and advanced into the aortic arch, with the telemeter body positioned in a subcutaneous pocket on the right flank. After a one-week recovery period, recordings began on the morning of the eighth day, with 10-second samplings every two minutes for at least three days for each animal. Radio signals were processed using a model RPC-1 receiver, a 20-channel data exchange matrix, APR-1 ambient pressure monitor, and a Data Quest ART Silver 2.3 acquisition system (Data Sciences International, St.Paul, MN, USA). The recording room was maintained at 21°C to 22°C with a 12-hour light:12-hour dark cycle [20].

### Glomerular filtration (GFR) measurements

The GFR of conscious mice was measured by fluorescein isothiocyanate (FITC)-labeled sinistrin clearance (a gift from Dr. Schock-Kusch, University of Heidelberg, Germany) after a single retro-orbital injection and consecutive blood sampling from the tail vein, as described previously [20].

### Plasma and urine analysis

Urinary osmolarity was determined using the freezing-point depression method [21]. Plasma and urine electrolytes were determined by standard clinical chemistry methods (IDEXX Veterinary Services, Ludwigsburg, Germany).

### Measurement of plasma renin concentration by radioimmunoassay

For measurements of plasma renin concentrations, blood was collected from conscious mice by puncturing the submandibular vessels with a 19-G needle. Approximately 20 µl of blood were collected into an ethylenediaminetetraacetic acid (EDTA)-containing microhematocrit tube. Red cells and plasma were separated by centrifugation; the plasma was kept frozen until renin measurements. Renin activity was measured by radioimmunoassay using a 20-fold dilution of 12 μl of plasma, (DiaSorin, Stillwater, MN, USA) as generation of Ang I following the addition of excess exogenous rat substrate (plasma renin concentration, PRC), with final plasma dilutions varying between 1:500 and 1:1,000. Ang I generation was determined for a three-hour incubation period at 37°C and expressed as an hourly average. In each assay, substrate without plasma was incubated for the same time, and any background Ang I formation was subtracted from the plasma-containing samples. In addition, background Ang I levels were determined in a plasma aliquot kept frozen without the addition of substrate until assaying [22].

### Arap1 expression analysis

The Arap1 mRNA was determined using quantitative RT-PCR (Light Cycler System, Roche, Mannheim, Germany) as described in detail recently [18].

### Cell culture experiments

Studies were performed in cultured rat glomerular mesangial cells (a gift from Dr. Eberhardt, Johann Wolfgang Goethe-Universität, Frankfurt am Main, Germany). This cell line expresses both Arap1 and the angiotensin receptors AT1 and AT2. The cells were grown as described previously [23]. Briefly, rat glomerular mesangial cells were cultured in RPMI 1640 (PAN-Biotech, Aidenbach, Germany) supplemented with 10% fetal calf serum (FCS), penicillin-streptomycin (100 U/ml, 100 μg/ml, Biochrom, Berlin, Deutschland), and 0.66 U/ml insulin (Sigma-Aldrich, Munich, Germany) at 37°C and 5% CO_2_. One day before the experiments, the FCS was reduced to 0.5% and penicillin-streptomycin was omitted from the medium. To address the effect of pro-inflammatory cytokines on Arap1 expression, interleukin-1 (50 ng/ml), TNF-α (100 ng/ml), or interferon- γ (IFN-γ) (100 ng/ml) was added to the wells. After 20 hours of incubation, the cells were washed with PBS, suspended in TRIzol reagent and stored at −80°C until further use. Unless it is otherwise stated, six wells of cells were investigated for each experimental condition.

### Isolated perfused mouse kidney

Mice were anesthetized with an intraperitoneal injection of 12 mg/kg xylazine and 80 mg/kg ketamine-HCl. The abdominal aorta was cannulated, and the right kidney was excised, placed in a thermostated moistening chamber (37°C), and perfused at constant pressure (100 mmHg). Finally, the renal vein was cannulated, and samples of the venous perfusate were taken every three minutes for determination of renin activity, as described above. The basic perfusion medium consisted of a modified Krebs-Henseleit solution, supplemented with 6 g/100 ml BSA and human red blood cells (10% hematocrit). The perfusate flow was monitored during the experiment. To assess the effect of angiotensin II on the renal perfusate flow and renin secretion, angiotensin II was added to the perfusate to a final concentration of 0, 10, 30, 100, 300 and 1,000 pM.

### Statistics

Data are expressed as the means and SEM. Statistical comparisons were made by Student's *t*-test or by ANOVA with Bonferroni *post hoc *tests when necessary.

## Results

### Blood pressure in Arap1-/- and +/+ mice during LPS-induced sepsis

**Figure 1:**
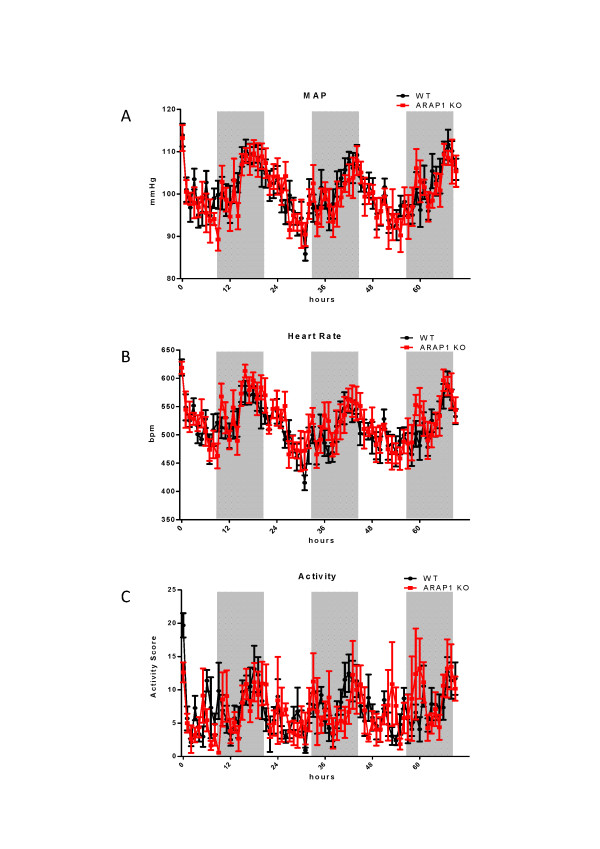
**Mean arterial blood pressure (MAP), heart rate and activity score for angiotensin receptor-associated protein (Arap)1-/- and wildtype mice**. MAP, heart rate and activity score were recorded by radio-telemetry for three consecutive days (*n *= 5 each). Shaded areas indicate light-off periods.

To address the relevance of Arap1 for vascular resistance and blood pressure regulation, cardiovascular parameters were measured in the Arap1-/- and wildtype mice using radio-telemetry over a period of 72 h (*n *= 5). As shown in Figure 1, the arterial blood pressure and activity scores under baseline conditions showed a circadian rhythm for both genotypes, with phases of activity during the light-off period and resting during the light-on period (12 hour:12 hour rhythm). Mean arterial pressure (MAP) averaged 102.8 ± 2.0 vs. 101.6 ± 1.6 mmHg (*P *= 0.66) during the light-off period, and 98.7 ± 1.9 vs. 98.3 ± 1.4 mmHg (*P *= 0.87) during the light-on period for wildtype mice vs. Arap1-/-. Similarly, the wildtype and Arap1-deficient mice did not differ in their systolic blood pressure, diastolic blood pressure, heart rate and activity score.

**Figure 2:**
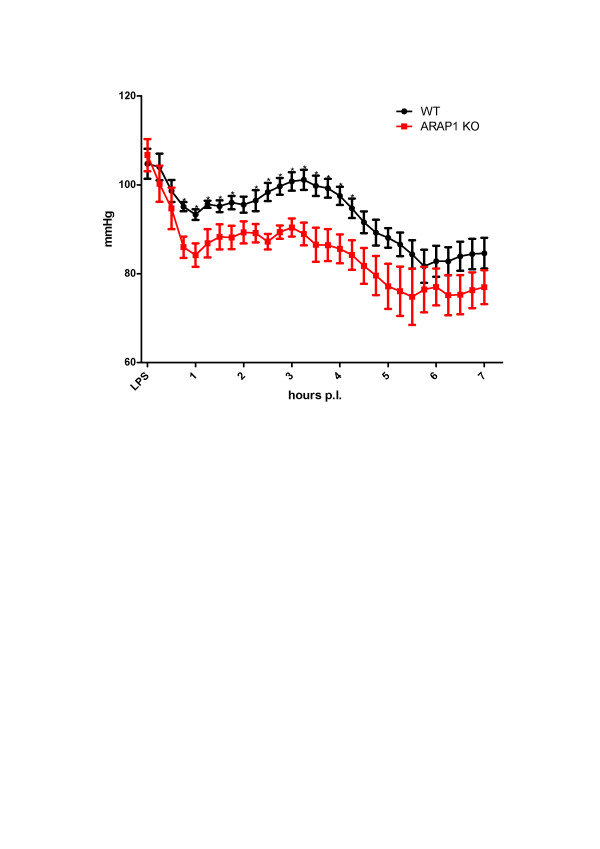
**Mean arterial blood pressure (MAP) in wildtype and angiotensin receptor-associated protein (Arap)1 -/- mice during endotoxemia**. MAP was measured by radio-telemetry following a single i.p. injection of lipopolysaccharide (LPS) (3 mg/kg) (*n *= 10 animals for each genotype).

During endotoxemia-induced sepsis, the blood pressure of Arap1-/- and wildtype mice was measured for seven hours after a single LPS (3 mg/kg) injection using radio-telemetry (*n *= 10 each). The activity scores reflecting the movement of the mice over the receiver grid were low in both genotypes during the experiment (0.23 ± 0.09 vs. 0.32 ± 0.17 for Arap1-/- and +/+ mice, respectively; *P *= 0.60). As shown in the tracings in Figure 2, one hour after LPS injection (3 mg/kg), the MAP dropped significantly in both genotypes, averaging 100.3 ± 4.1 vs. 84.1 ± 2.6 mmHg for the Arap1-/- mice pre- and post-sepsis, respectively (*P *= 0.001), and 104.0 ± 3.0 mm vs. 93.3 ± 1.2 Hg for the wildtype mice pre- and post-sepsis, respectively (*P *= 0.013). Thus, the MAP fell to significantly lower values in the Arap1-/- compared with the values in the wildtype mice (*P *= 0.006). During the following two hours, MAP partly recovered in both genotypes, but the values remained significantly lower in the Arap1-/- than in the wildtype mice (90.4 ± 2.0 vs. 101.1 ± 2.3 mmHg; *P *= 0.002). Between five and six hours after LPS injection, the MAP reached a minimum, with similar values in Arap1-/- (74.8 ± 6.0 mmHg) and wildtype mice (76.7 ± 3.7 mmHg; *P *= 0.35).

### Blood pressure during LPS-induced sepsis and concomitant RAS blockade

**Figure 3:**
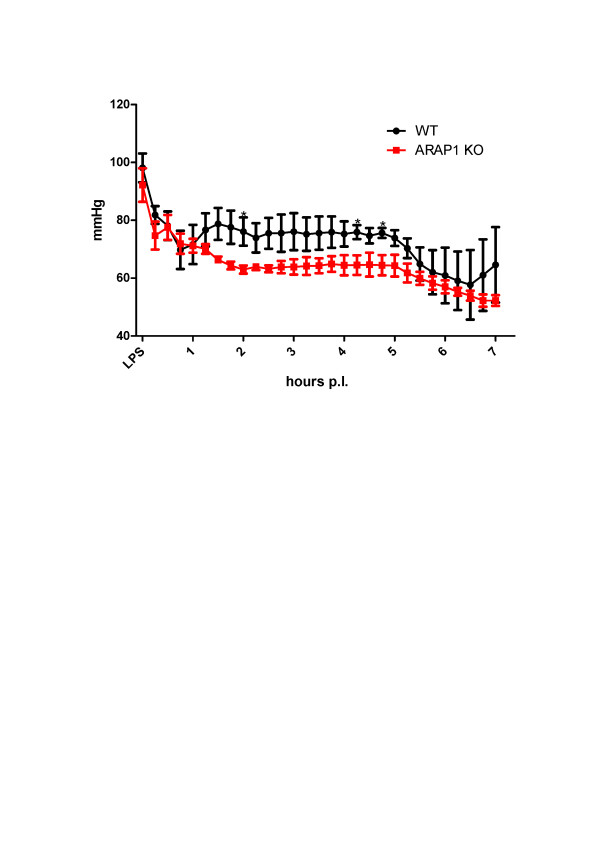
**Mean arterial blood pressure (MAP) during endotoxemia in angiotensin converting enzyme (ACE) inhibitor-pretreated mice**. Wildtype and angiotensin receptor-associated protein (Arap)1 -/- mice were pretreated with the ACE inhibitor enalapril (10 mg/kg/d) (*n *= 5 animals for each genotype).

Because of the MAP differences between the Arap1-/- and +/+ mice, we assessed the impact of RAS activation on blood pressure regulation during sepsis-induced hypotension. Before the injection of LPS, mice were pretreated with the ACE-inhibitor enalapril (10 mg/kg/d) for three consecutive days. Enalapril reduced the MAP in both genotypes. MAP before LPS injection was numerically lower in the enalapril-pretreated Arap1-/- mice than in the wildtypes, without reaching levels of significance (84.3 ± 1.9 vs. 90.7 ± 3.4 mmHg in Arap1-/- and +/+ mice, respectively; *n *= 5; *P *= 0.17) whereas systolic blood pressure was significantly lower in Arap1-/- mice (103.1 ± 1.0 vs. 109.5 ± 2.2 for Arap1-/- and +/+, respectively; *P *= 0.048). Two hours after the LPS injection, the MAP in the wildtype mice fell to 76 ± 4.0 mmHg and reached a plateau phase before decreasing to a minimum of 57.7 ± 9.8 mmHg 6.5 hours after LPS injection (Figure 3). Thus, enalapril blunted the transient blood pressure recovery that was observed in the previous experiment in the absence of enalapril. In the Arap1-/- mice, the MAP two hours after the LPS injection was significantly lower than in the wildtype mice (63.0 ± 1.3 vs. 76.0 ± 4.0 mmHg; *P *= 0.04). The MAP levels in the Arap1-/- mice subsequently continued to fall to a minimum at 6.5 hours after the LPS injection (54.0 ± 1.5 mmHg), not different from the levels in the wildtypes (57.7 ± 9.8 mmHg) (Figure 3).

In summary, during endotoxemia the blood pressure homeostasis in the Arap1-deficient mice was more severely compromised than in wildtype mice. The activation of the RAS during LPS-induced sepsis apparently supported a partial and transient recovery of the blood pressure.

### Expression of Arap1 during LPS-induced sepsis: in vivo and in vitro experiments

**Figure 4:**
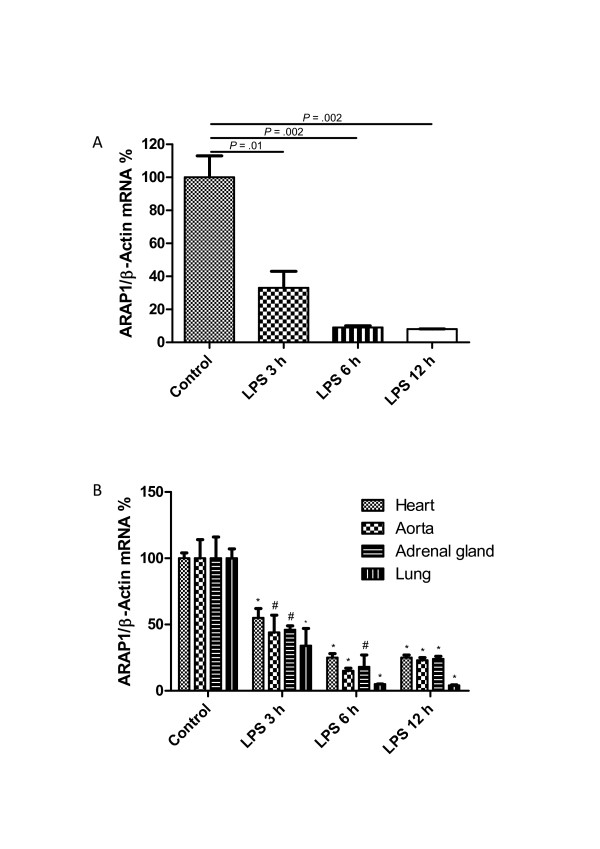
**Angiotensin receptor-associated protein (Arap)1 mRNA expression after Lipopolysaccharide (LPS) injection (**A**) Renal Arap1 mRNA expression 0, 3, 6 and 12 hours after LPS injection determined by quantitative RT-PCR**. Values are given as relative units normalized to β-actin expression (*n *= 6 for each genotype). (**B**) Relative Arap1 mRNA expression 0, 3, 6 and 12 hours after LPS injection in the heart, aorta, adrenal gland and lung compared to vehicle-injected animals (*n *= 6 for each group). (#*P *<0.05 compared to control; *P <0.01 compared to control).

**Figure 5:**
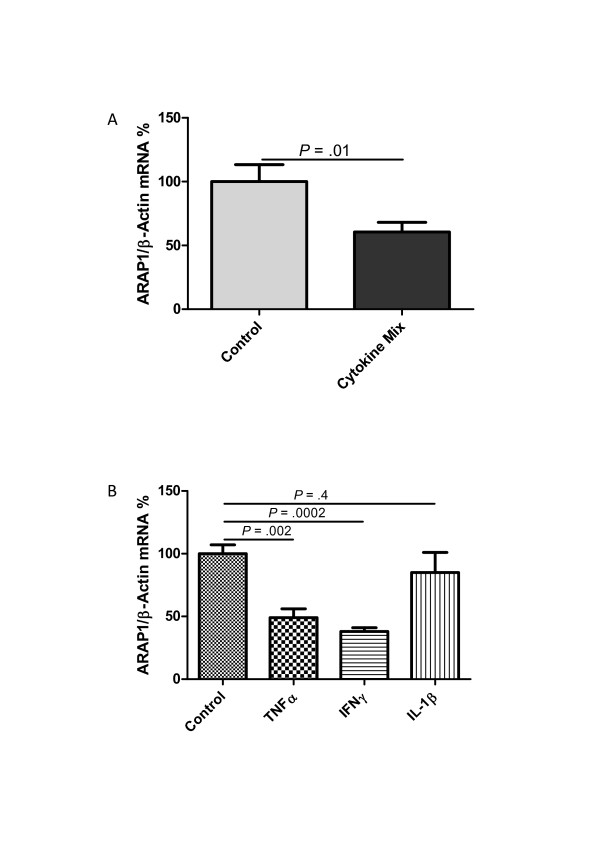
**Angiotensin receptor-associated protein (Arap)1 mRNA expression in cultured rat glomerular mesangial cells**. Arap1 mRNA expression was measured following incubation with (**A**) a cytokine mix containing IL-1β (50 ng/mL), tumor necrosis factor (TNF)-α (100 ng/mL) and interferon (IFN)-γ (100 ng/mL) or (**B**) single cytokines (interleukin (IL)-1β (50 ng/mL), TNF-α (100 ng/mL), IFN-γ (100 ng/mL).

Despite the activation of the RAS, hypotension associated with tissue hypoperfusion is a common symptom in septic patients. We hypothesized that changes in Arap1 expression under septic conditions may contribute to the blunted vascular sensitivity to angiotensin II during sepsis. Therefore, the Arap1 expression was determined in various organs of the mice following LPS injection. Two hours after LPS injection, the Arap1 mRNA expression in the kidney was significantly down-regulated to 59 ± 13% of the control levels and successively decreased to 33 ± 10%, 9 ± 1% and 8 ± 0.2% of the controls, 3, 6 and 12 hours after LPS-injection, respectively (Figure 4A). Similar results were obtained for other tissues, such as the heart, aorta, adrenal gland and lung (Figure 4B). To further assess the mechanism underlying the down-regulation of Arap1 expression during sepsis, additional *in vitro *studies were performed. The Arap1 expression in cultured mesangial cells of rats was determined following the incubation with pro-inflammatory cytokines. Incubation with a cytokine mix containing IL-1β (50 ng/mL), TNF-α (100 ng/mL) and IFN-γ (100 ng/mL) resulted in a 40 ± 7% decrease of the Arap1 mRNA expression compared with that of the controls (*P *= 0.01; Figure 5A). Similar results were obtained for the incubation of cultured mesangial cells with individual cytokines, such as TNF-α (49 ± 7% of the controls; *P *= 0.002) or IFN-γ (38 ± 3% of the controls; *P *= 0.0002). By contrast, the incubation with IL-1β alone did not alter the Arap1 mRNA expression (*P *= 0.4; Figure 5B).

### Vascular reactivity and renin secretion in vitro: isolated perfused kidney

**Figure 6:**
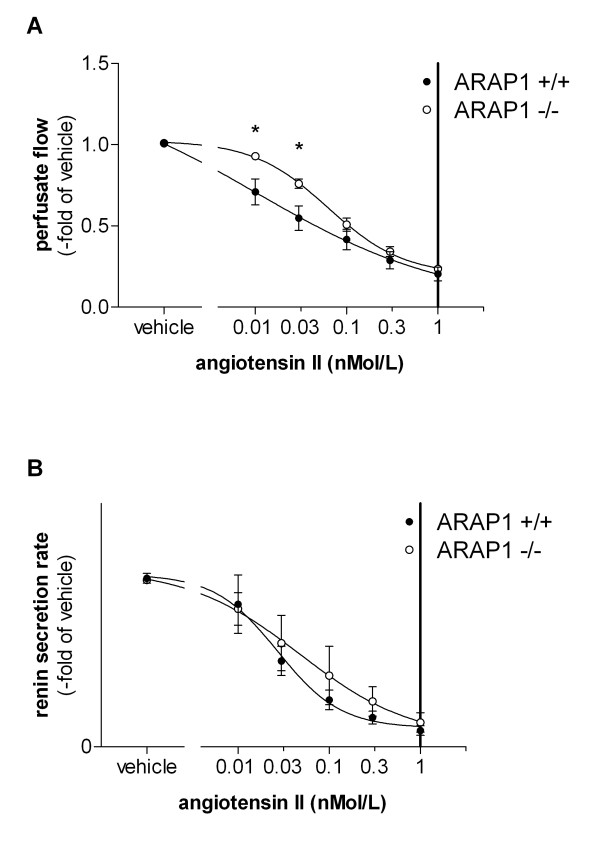
**Isolated perfused kidney (**A**) Perfusate flow at constant perfusion pressure of 100 mmHg in isolated perfused kidneys from angiotensin receptor-associated protein (Arap)1-/- and wildtype mice (*n *= 5)**. After pre-relaxation with isoproterenol (10 nM), angiotensin II was added to the perfusate to a final concentration of 10, 30, 100, 300 and 100 pM. For each concentration, three values were determined over a period of three minutes. (**B**) Renin secretion from isolated kidneys perfused according to the same protocol (*n *= 5).

Systemic blood pressure is controlled by multiple humoral and neuronal factors. Because of the unaltered blood pressure in the Arap1-/- and +/+ mice under baseline conditions and considering the multiple factors impinging on vascular resistance *in vivo*, we used the isolated perfused kidney model to assess the relevance of Arap1 for vascular reactivity in a controlled *in vitro *model. After pre-relaxation using the β1-adrenoreceptor agonist isoproterenol (10 nM), the renal perfusate flow during a constant perfusion pressure (100 mmHg) was reduced by angiotensin II in a dose-dependent manner, as shown in Figure 6A. The angiotensin II-induced changes in perfusate flow were right-shifted in isolated perfused kidneys from the Arap1-/- mice compared with those in the kidneys from the wildtype mice. Consequently, the renal vascular resistance for a given dose of angiotensin II was lower in the kidneys from the Arap1-/- than in those from the wildtype mice (Figure 6A). The most notable differences between Arap1-/- mice and wildtype controls were observed in the low physiological range from 10 to 100 pM (*n *= 5 each group; *P *<0.01 between genotypes for 10 pM and 30 pM). The maximum vascular responses (1,000 pM angiotensin II) were similar in both genotypes and resulted in a decrease of the perfusate flow by 80% (Figure 6A).

**Figure 7:**
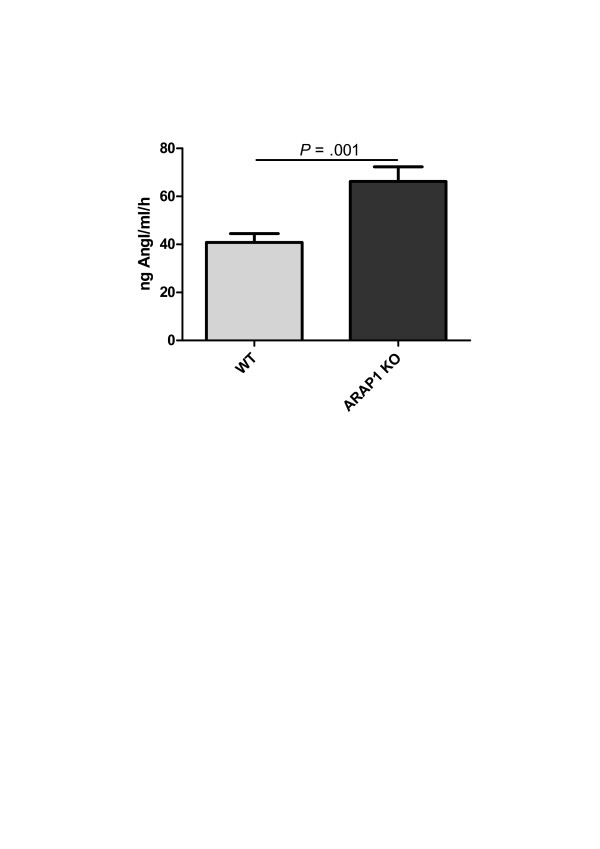
Plasma renin concentration in conscious angiotensin receptor-associated protein (Arap)1-/- and wildtype mice (*n *= 23 for each genotype).

Renin secretion from the isolated perfused kidneys was determined in parallel to the perfusate flow to address the renin secretory capacity of Arap1-/- and wildtype mice kidneys in the absence of modulating systemic factors. To mimic the *in vivo *stimulatory input of the sympathetic nervous system on renin secretion in the isolated kidney, isoproterenol (10 nM) was added to the perfusate. The renin secretion after stimulation with isoproterenol (10 nM) was markedly enhanced in kidneys from Arap1-/- mice compared with those from the wildtype mice (1,427 ± 318 vs. 667 ± 174 ng ang I/ml/h; *P *<0.001). By contrast, the relative suppression (percent of isoproterenol) of renin secretion by angiotensin II did not differ between the genotypes, suggesting an unaltered negative feedback function of angiotensin II (Figure 6B). Similarly, like in the isolated perfused kidney, the PRC *in vivo *was increased by 62% in Arap1-/- compared to wildtype mice. Thus, the PRC was 66.2 ± 6 in Arap1-/- and 40.8 ± 4 ng Ang I/ml/h in wildtypes (*n *= 23 per group; *P *= 0.001; Figure 7). Baseline parameters of renal function did not differ between the Arap1-/- and wildtype mice. Thus, GFR averaged 308 ± 13 and 301 ± 12 µl/min, in Arap1-/- and wildtype mice, respectively (*n *= 5; *P *= 0.68; not shown). Similarly, the plasma electrolyte concentrations and urine electrolyte:osmolality ratios did not differ considerably between the Arap1-/- and wildtype mice, as summarized in Table 1.

**Table 1:** Panel of plasma electrolytes and urine electrolyte/osmolality ratios in Arap1-/- and wildtype mice.

Electrolytes		WT	Arap1 KO	*P*-value
Plasma	Na (mmol/l)	151.4 ± 2.0	154.3 ± 1.7	0.30
	
	K (mmol/l)	5.10 ± 0.16	5.23 ± 0.19	0.64
	
	Cl (mmol/l)	109.0 ± 1.7	109.9 ± 1.4	0.71

Urine	Na (mmol*kg/mosmol*l)	0.05 ± 0.009	0.051 ± 0.006	0.88
	
	K (mmol*kg/mosmol*l)	0.079 ± 0.004	0.080 ± 0.002	0.92
	
	Cl (mmol*kg/mosmol*l)	0.085 ± 0.003	0.097 ± 0.006	0.17

*n *= 6 for each genotype. Arap1, angiotensin receptor-associated protein 1

## Discussion

Arap1 is a protein that interacts with the angiotensin II AT1 receptor, and *in vitro *studies suggested that Arap1 facilitates the surface expression of the AT1 receptor and, hence, acts as a positive local regulator of AT1 receptor function [17]. In the present study, we used Arap1-deficient mice to investigate the possible involvement of Arap1 in sepsis-induced hypotension. We hypothesized that a dysregulation of Arap1 expression during sepsis may be involved in the hyporeactivity of vascular AT1 receptors, contributing to a decrease in the total vascular resistance.

Vascular hypo-responsiveness to pressor substances, such as angiotensin II contributes to hypotension in septic patients [1]. Arap1 was shown to enhance AT1 receptor surface expression in cultured cells [17], a result that was functionally confirmed at the whole organ level in our study. Therefore, we hypothesized that Arap1-dependent modulation of the AT1 receptor may contribute to angiotensin resistance during sepsis. In fact, the Arap1 expression in several organs declined during the course of the experimental sepsis, reaching levels below 10% of the baseline. This down-regulation of Arap1 expression could be recapitulated *in vitro*, when cultured mesangial cells were exposed to pro-inflammatory cytokines, such as TNFα and IFNγ. Thus, the reduced Arap1 expression during sepsis may be mediated by pro-inflammatory cytokines, all of which are known to be markedly elevated during endotoxemia [23,24]. Furthermore, Arap1 abundance has been shown to be dependent on angiotensin II levels, with angiotensin II suppressing Arap1 expression; the regulation of Arap1 expression was related to changes in transcriptional activity, because Arap1 mRNA and protein abundances changed in parallel under all conditions [18]. Plasma angiotensin II concentrations were shown to be 5-fold increased six hours after the induction of endotoxemia by a single LPS injection in rats [23], and PRA was elevated up to 25-fold in septic human patients [5]. Thus, angiotensin II in combination with pro-inflammatory cytokines likely accounts for the down-regulation of Arap1 during LPS-induced sepsis. The virtual loss of Arap1 during endotoxemia would reduce the surface expression of the AT1 receptor in the vasculature and would contribute to the hyporesponsiveness to angiotensin II observed during sepsis. With the endotoxemia-triggered down-regulation of Arap1 to levels below 10% of the control level, the wildtype mice approximated the Arap1-deficient mice. Thus, despite the marked blood pressure differences between the Arap1+/+ and -/- mice during the early time course of endotoxemia, blood pressure was indistinguishable between the genotypes when the blood pressure nadir was reached, approximately six hours after the LPS administration. At this particular time, the Arap1 expression in the wildtype mice was as low as 9% of the baseline abundance, similar to the situation in the Arap1-/- mice.

In contrast to the baseline conditions, when the MAP was maintained in the Arap1-/- mice, the fall in blood pressure after the induction of endotoxemia was more pronounced in the Arap1-/- than in the wildtype mice, at least before the minimum values were reached after approximately six hours. Apparently, when the RAS is stimulated, and when increased concentrations of angiotensin II are required to maintain the MAP, the relevance of Arap1 for AT1 receptor surface expression is unmasked. Our experiments with mice pre-treated with enalapril further suggest that blood pressure maintenance is more dependent on intact AT1 receptor activity during sepsis than under normal conditions. Thus, enalapril reduced the baseline MAP from 104.9 to 98 mmHg in the wildtype mice and from 106.8 to 92 mmHg in the Arap1-/- mice before the LPS administration, a difference of 6.9 and 14.8 mmHg, respectively. However, six hours after the induction of endotoxemia, the blood pressure differences caused by enalapril increased to -19 in the Arap1-/- and to -21 mmHg in the wildtype mice, with no difference between the genotypes, suggesting that the relative contribution of angiotensin II to maintaining MAP had increased.

A closer inspection of the time course of the MAP after LPS injection revealed a transient recovery of the blood pressure approximately 2.5 hours after the induction of endotoxemia. This transient recovery has been suggested to be related to an activation of the RAS and an increased endothelin production [25]. Our data are consistent with this assumption because the transient increase in MAP was blunted in the presence of enalapril in both genotypes, suggesting that an activation of the RAS predominantly accounts for the temporary stabilization of blood pressure. It should be noted that the Arap1 expression at this time point was only slightly reduced when compared with the expression during the later course of endotoxemia.

Although down-regulation of Arap1 during endotoxemia appears to be relevant for the development of hypotension and, by inference, hyporesponsiveness to angiotensin II, it should be noted that other mechanisms, such as inadequate formation of vasodilator agents and resistance to other vasoconstrictors, are relevant in the pathogenesis of septic circulatory failure [1]. The contribution of these single mechanisms to the overall dysregulation of vascular tone may vary during the course of the disease.

Experiments in isolated perfused kidneys, used as a model of organ vascular resistance, revealed that the sensitivity of vascular AT1 receptors to angiotensin II was reduced in the Arap1-/- kidneys, which is consistent with previous *in vitro *data indicating that Arap1 enhances the membrane surface expression of the AT1 receptor [17,26]. The basal renal vascular resistance, that is, in the absence of angiotensin II, was indistinguishable between the isolated kidneys from the Arap1-/- and the wildtype mice, indicating that the angiotensin II-independent components of the vascular tone were unaltered in Arap1-deficient kidneys. The dose-response curve of vascular resistance vs. angiotensin II concentration in the isolated perfused kidney closely mimics the *in vivo *situation. Thus, the steepest slope of the dose-response curve was observed in the angiotensin II concentration range of 30 to 100 pM, similar to concentrations *in vivo*, which have been estimated to be in the range of 50 to 100 pM for the mouse [27].

Despite the different vascular AT1 receptor sensitivities in the Arap1-/- and wildtype mice *in vitro*, the baseline blood pressure in the Arap1-/- mice was inconspicuous, suggesting that corresponding changes in systemic vasopressor systems compensated for the deviation of vascular sensitivity to angiotensin II. In fact, the PRC was increased by 60% in the Arap1-/- mice compared with that in the wildtype mice, which apparently allowed for full restoration of the total vascular resistance to wildtype levels, at least under baseline conditions. When the Arap1-/- mice were treated with an ACE inhibitor, the systolic blood pressure decreased to a larger extent than in the wildtype mice, suggesting again that blood pressure maintenance in the Arap1-/- mice requires an activated RAS. A compensatory stimulation of RAS has been shown for several models of compromised AT1 receptor function, such as AT1_a,b _receptor-deficient mice [28]. In the situation of complete AT1 deficiency, the basal PRC is elevated 6- to 10-fold compared with that in control animals; this increase in PRC has been attributed to a disinhibition of renin secretion because of an interruption of the direct negative feedback loop of angiotensin II on the renin-secreting cells and to systemic effects, such as low arterial pressure. Activation of the RAS in the Arap1-/- mice, however, appears to be related to the systemic rather than direct effects of angiotensin II on renin-producing cells because the relative suppression of renin secretion by angiotensin II was preserved in the isolated perfused mouse model. These functional data are consistent with the results of a recent localization study that suggested that renin-producing cells of the afferent arterioles do not express Arap1 [18].

## Conclusion

Septic patients frequently suffer from compromised sensitivity of the vasculature to pressor hormones, such as angiotensin II. Our *in vivo *and *in vitro *data suggest that Arap1 acts as a positive modulator of vascular AT1 receptors. During endotoxemia, Arap1 expression is markedly down-regulated and sepsis-induced circulatory failure is aggravated in Arap1-deficient mice. Loss of Arap1 function during endotoxemia therefore may contribute to the vascular hyporeactivity to angiotensin II in septic patients.

## Key messages

• Septic patients frequently suffer from compromised sensitivity of the vasculature to pressor hormones, such as angiotensin II.

• The AT1 receptor-associated protein Arap1 increases vascular sensitivity to angiotensin II.

• In endotoxemia, Arap1 expression is markedly down-regulated.

• Arap1 deficiency aggravates sepsis-induced circulatory failure.

• Down-regulation of Arap1 during endotoxemia may contribute to vascular hyporeactivity to angiotensin II during endotoxemia.

## Abbreviations

ACE: angiotensin converting enzyme; Arap1: angiotensin receptor-associated protein 1; Bp: blood pressure; EDTA: ethylenediaminetetraacetic acid; FCS: fetal calf serum; FITC: fluorescein isothiocyanate GFR: glomerular filtration rate; IFNγ: interferon γ; LPS: lipopolysaccharide; MAP: mean arterial pressure; PBS: phosphate-buffered saline; PRC: plasma renin concentration; RAS: renin angiotensin-system; TNFα: tumor necrosis factor α

## Competing interests

The authors declare that they have no competing interests.

## Authors' contributions

KMe, FS, KH, OY and HC designed the study. KMe, VK, KMi and OY performed the animal experiments and analyzed the data. FS carried out the isolated perfused kidney experiments. ED performed the *in vitro *studies. KMe and HC drafted the manuscript. All authors participated in revising the manuscript. HC finalized the manuscript. All authors read and approved the final manuscript.
